# Self‐regulated learning in the clinical context: a systematic review

**DOI:** 10.1111/medu.13615

**Published:** 2018-06-25

**Authors:** Maaike A van Houten‐Schat, Joris J Berkhout, Nynke van Dijk, Maaike D Endedijk, A Debbie C Jaarsma, Agnes D Diemers

**Affiliations:** ^1^ Department of General Practice and Elderly Care Medicine University Medical Centre Groningen University of Groningen Groningen the Netherlands; ^2^ Centre for Research and Innovation in Medical Education University Medical Centre Groningen University of Groningen Groningen the Netherlands; ^3^ Centre for Evidence‐Based Education Academic Medical Centre University of Amsterdam Amsterdam the Netherlands; ^4^ Department of General Practice Academic Medical Centre University of Amsterdam Amsterdam the Netherlands; ^5^ Department of Educational Sciences Faculty of Behavioural, Management and Social Sciences University of Twente Enschede the Netherlands

## Abstract

**Objectives:**

Research has suggested beneficial effects of self‐regulated learning (SRL) for medical students' and residents' workplace‐based learning. Ideally, learners go through a cyclic process of setting learning goals, choosing learning strategies and assessing progress towards goals. A clear overview of medical students' and residents' successful key strategies, influential factors and effective interventions to stimulate SRL in the workplace is missing. This systematic review aims to provide an overview of and a theoretical base for effective SRL strategies of medical students and residents for their learning in the clinical context.

**Methods:**

This systematic review was conducted according to the guidelines of the Association for Medical Education in Europe. We systematically searched PubMed, EMBASE, Web of Science, PsycINFO, ERIC and the Cochrane Library from January 1992 to July 2016. Qualitative and quantitative studies were included. Two reviewers independently performed the review process and assessed the methodological quality of included studies. A total of 3341 publications were initially identified and 18 were included in the review.

**Results:**

We found diversity in the use of SRL strategies by medical students and residents, which is linked to individual (goal setting), contextual (time pressure, patient care and supervision) and social (supervisors and peers) factors. Three types of intervention were identified (coaching, learning plans and supportive tools). However, all interventions focused on goal setting and monitoring and none on supporting self‐evaluation.

**Conclusions:**

Self‐regulated learning in the clinical environment is a complex process that results from an interaction between person and context. Future research should focus on unravelling the process of SRL in the clinical context and specifically on how medical students and residents assess their progress towards goals.

## Abstract 2

This study outlines the complexity of self‐regulated learning as a process with individual differences between students that is influenced by multiple factors at individual, social and contextual levels.

## Introduction

Because of the rapid pace of developments in medical science, it is important for medical students and residents to learn how to practise lifelong learning as doctors. One important strategy for the lifelong professional development of medical students and residents is self‐regulated learning (SRL).^1^, ^2^, ^3^ In the field of education research, many models have been developed to describe the process of SRL.^4^, ^5^, ^6^, ^7^ Overall, SRL refers to the modulation of affective, cognitive and behavioural processes throughout a learning experience in order to reach a desired level of achievement.^5^, ^8^ A meta‐analysis by Sitzmann and Ely showed that the various processes described by different theories of SRL can be categorised into the following three types: regulatory agent (goal setting); regulatory mechanism (e.g. planning, monitoring, learning strategies, motivation and emotion control), and regulatory appraisal (self‐evaluation, attributions and self‐efficacy).^9^ Multiple studies have shown the positive effects of students' use of self‐regulation strategies in academic outcomes.^10^, ^11^

However, a large part of medical education occurs in the workplace instead of in classrooms.^3^, ^12^, ^13^, ^14^, ^15^ In workplace‐based learning, medical students and residents learn from their experiences in clinical practice.^12^ Self‐regulated learning in a clinical context is different from SRL in an academic setting because in a clinical context the student cannot solely focus on his or her own learning goals as these are subsidiary to the provision of health care to patients.^16^ It may therefore be that students who adequately self‐regulate their learning in an academic setting may have difficulties in self‐regulating their learning in the more complex clinical setting. To maximise the learning potential of workplaces, medical education programmes increasingly include elements to promote SRL in the clinical environment, such as reflective learning, the setting of learning goals and the use of portfolios.^3^, ^17^, ^18^ It is important to better understand *how* medical students and residents regulate their learning in the clinical environment in order to successfully support the development of medical students' and residents' SRL skills.

Considering the suggested beneficial effects of SRL in academic settings, we aimed to determine how medical students and residents regulate their learning in the clinical environment. More specifically, our research questions were: (i) What theoretical models have been used to study medical students' and residents' SRL in the clinical environment? (ii) What SRL strategies do medical students and residents use in the clinical environment? (iii) Which factors of SRL have been reported to influence medical students and residents? (iv) What interventions have been used to support SRL in medical students and residents and what effects did the interventions have?

## Methods

We conducted a systematic review according to the guidelines of the Association for Medical Education in Europe (AMEE).^19^

### Selection process

We systematically searched for relevant publications describing how undergraduate medical students and/or postgraduate medical residents regulate their learning in the clinical environment. Although the terms ‘SRL' and ‘self‐directed learning' (SDL) have some similarities and differences, these terms are not clearly distinguished in the literature and are often used interchangeably.^20^ We therefore used both terms in our search strategy. A research librarian helped us to design the search strategy. The first author systematically searched six databases: PubMed; EMBASE; Web of Science; PsycINFO; ERIC, and the Cochrane Library. The search was performed in July 2016 and the terms used to search titles and abstracts were:

1. self‐regulat*, SRL, self‐direct*, SDL, learn*;
2. resident, residents, intern, interns, trainee*, postgraduate student*, postgraduates, medical student*, clinical clerk, clinical clerks, undergraduate student*, undergraduates, and
3. postgraduate education, graduate education, medical education, clinical education, health profession* education, postgraduate train*, graduate train*, medical train*, clinical train*, postgraduate learn*, internship, residency, undergraduate education, undergraduate learn*, undergraduate train*, medical school, clinical placement, medical curriculum, clinical apprenticeship, clinical clerkship.

The search identified 3341 articles. Original studies were included in the review if: (i) the main purpose of the research focused on SRL or SDL in the clinical workplace, and (ii) participants were undergraduate medical students in their clerkships or postgraduate medical residents. We excluded studies focusing on an educational setting other than the medical education setting (e.g. nursing or dentistry) and further limited our search to articles published between 1992 and 2016. We also excluded articles in a language other than English or Dutch.

In addition to the electronic search, we conducted hand searches of titles and abstracts from January 2012 through to October 2016 in the following journals: *Medical Education*;* Advances in Health Sciences Education*;* Medical Teacher*, and *Academic Medicine*. Finally, we reviewed the bibliographies of the included articles to identify other relevant articles.

### Review procedure

First, four reviewers (MAvH‐S, JJB, NvD and ADD) independently selected articles based on the title and abstract and decided whether the inclusion criteria were met. The reviewers then worked in pairs and reached a mean agreement between pairs of 93.2%. If there was disagreement between the reviewers, we decided to take the article to the next stage for evaluation of eligibility through full text reading. Two reviewers (MAvH‐S and ADD) performed full text reading and reached an agreement level of 94.3%. Disagreements were resolved by discussion and by consulting the other authors.

Finally, two reviewers (MAvH‐S and ADD) independently assessed the methodological quality of the included studies using the Medical Education Research Study Quality Instrument (MERSQI)^21^ for experimental, quasi‐experimental and observational studies and the consolidated criteria for reporting qualitative studies (COREQ)^22^ for qualitative studies. Discrepancies between assigned points were resolved by discussion until consensus was reached. The overall level of agreement was 95.0%.

## Results

**Figure 1:**
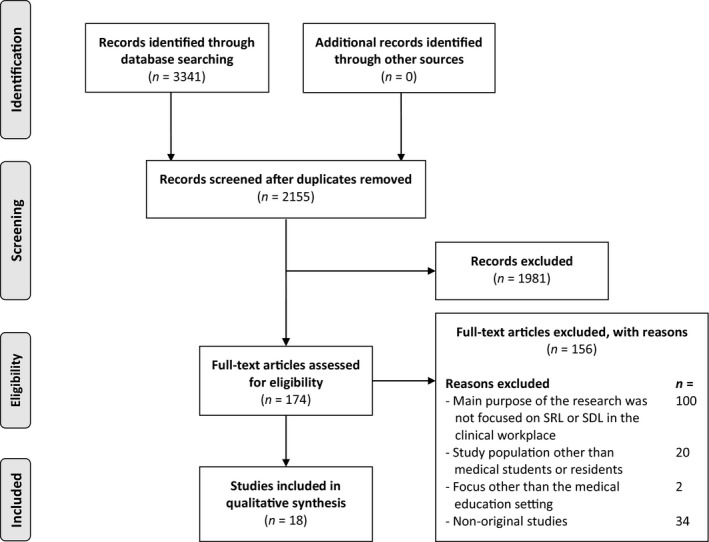
Study selection process. SDL = self‐directed learning; SRL = self‐regulated learning

The study selection process for this review is depicted in Fig. 1. In the final analyses, we included 18 studies: 10 observational studies; six qualitative studies, and two mixed‐methods studies. The observational studies all consisted of survey data. Of the qualitative studies, three studies conducted interviews, two studies conducted focus groups, and one study conducted both interviews and focus groups. The two mixed‐methods studies performed either interviews or focus groups in combination with survey data. In eight of the studies, the research population consisted of medical students in the clinical environment. The remaining 10 studies involved residents. Two of these studies also investigated the perspectives of their programme directors and two others added the perspectives of faculty members. A detailed description of the included studies is presented in Table S1. The outcome of quality assessment of the qualitative studies was reasonably good and of the quantitative studies was moderate (Tables S2 and S3).

The results of the review are structured according to the different research questions. Most studies addressed more than one question.

### What theoretical models have been used to study medical students' and residents' SRL in the clinical environment?

Of the 18 studies we included, seven studies^2^, ^3^, ^23^, ^24^, ^25^, ^26^, ^27^ reported the cyclical three‐phase model of Zimmerman,^8^ two studies^28^, ^29^ reported the theoretical model of Knowles,^30^ and one study^31^ used a theoretical framework based on the principles of Pintrich.^32^ Although they differ in their perspectives, these three theories agree upon active engagement, goal setting, implementation of a plan and self‐evaluation of the process.^20^, ^33^

Although the remaining eight studies^34^, ^35^, ^36^, ^37^, ^38^, ^39^, ^40^, ^41^ in this review did not describe a specific theoretical model, they did mention the same processes (goal setting, implementation of a plan and self‐evaluation) when defining SRL.

### What SRL strategies do medical students and residents use in the clinical environment?

Three studies addressed this question, each with a different approach.

Firstly, Berkhout et al.^23^ described five different patterns of clinical students' self‐assessed SRL behaviour: engaged (hardworking, motivated and not afraid to make mistakes); critically opportunistic (unstructured learning behaviour, critical of the learning environment, loses motivation easily); uncertain (passive behaviour, dependent on supervisor, requires a safe environment in which to learn); restrained (highly motivated and self‐critical and afraid to ask questions), and effortful (hard working, needs to be told what to do, and afraid to admit being in difficulty). The authors noticed a great difference in patterns regarding goals, metacognition, communication, effort and dependence on external regulation for learning. Results also showed that the difficulty of learning in a clinical context was reflected in all these SRL patterns by the little‐planned SRL behaviour and limited goal setting.

Secondly, Woods et al.^24^ identified three distinct approaches to SRL in the clinical environment. The first approach, ‘acquiescing to a perceived lack of learning opportunities', emphasised the systemic and environmental barriers that medical students perceived. In the second approach, ‘choosing from available opportunities', students focused on the balance of different learning needs and realised that some time must be spent on service rather than learning. The last approach was ‘creating new learning opportunities', in which students tried to optimise their amount of learning. The three approaches found by Woods et al.^24^ relate to four of the patterns described by Berkhout et al.^23^ Only the pattern of uncertainty was not mentioned by the students in the study by Woods et al.^24^

Finally, Sagasser et al.^3^ conducted research on the self‐regulatory activities of family practice residents. The authors identified that self‐regulation of learning occurred in a short and a long loop. Residents used short‐loop regulation when faced with relatively simple problems and long‐loop self‐regulation in more complex problems and during longer periods. Both regulation loops were regulated internally; however, the long self‐regulation loop could also be affected externally.

### Which factors of SRL have been reported to influence medical students and residents?

Seven of the included studies addressed the factors influencing SRL. The results are summarised in Table 1. We structured the influential factors according to the categories described in the literature,^4^, ^5^ without giving weight to the different categories: individual (factors describing characteristics of the individual learner); contextual (factors describing characteristics of the clinical context), and social (factors describing influences of other actors in the learning process).

**Table 1:** Factors influencing the self‐regulated learning of medical students and residents in the clinical environment

Individual	Contextual	Social
Intrinsic motivation^2^, ^3^, ^29^ (to become a good doctor) Attention focusing^2^, ^3^ Goal‐setting skills^24^, ^26^, ^29^, ^40^, ^41^ Reflective skills^29^, ^41^ Experienced difficulty in the task^2^ Confidence in self‐directed learning abilities^2^, ^40^ Plan development and implementation^3^, ^41^ Previous experiences^2^	Available time^2^, ^3^, ^24^, ^29^ (time pressure) Characteristics of the learning environment^2^, ^3^, ^29^ (work climate, engagement in team) Patient‐related factors^2^, ^3^, ^26^, ^29^, ^40^ (patient encounters, types of patient)	Influence of supervisor^3^, ^23^, ^26^, ^29^Faculty staff support^26^, ^40^Familiarity with other people present in a certain setting^2^

Most studies reported motivation and goal‐setting skills as positive individual influential factors.^2^, ^3^, ^24^, ^26^, ^29^, ^40^, ^41^ Some studies^2^, ^9^ have shown that goal setting could have a motivational function and that goals, therefore, can function as regulatory agents for SRL. Individual barriers to achieving learning goals were difficulty with personal reflection, difficulty with goal generation, and problems with plan development and implementation.^41^ Other individual barriers to SRL were concentration problems, difficulty with a task, and undertaking too many tasks at the same time.^3^ One of the most frequently mentioned positive contextual factors was patient care. Patient contacts stimulate students' and residents' SRL as a starting point for learning.^2^, ^3^, ^26^, ^29^, ^40^ Almost all studies reported time pressure as a contextual barrier to SRL.^2^, ^3^, ^24^, ^29^, ^41^ Positive social influential factors were the influences of a coach or a supervisor and peers.^2^, ^3^, ^26^, ^29^

### What interventions have been used to support SRL in of medical students and residents? What effects did the interventions have?

Nine studies addressed this question,^27^, ^28^, ^34^, ^35^, ^36^, ^37^, ^38^, ^39^, ^40^ and described three types of intervention: (i) guiding of SRL via mentoring or coaching; (ii) support of SRL via learning plans and goal setting, and (iii) supportive tools, such as an online environment and clinical encounter cards.

Two studies showed that a *mentor‐guided* SRL approach affected residents' practice in a positive way.^28^, ^36^ In the study performed by George et al*.,* 36 residents demonstrated progressive independence in setting learning goals. Aho et al.^28^ examined whether a mentor‐guided SRL approach improved practice habits among surgery residents. Results showed that all residents displayed improvement in all practised tasks. Residents also reported that the frequency of practising was higher in this mentor‐guided SRL rotation. In addition, 50% of the residents reported that their skills had improved compared with their peers. Thus, these studies revealed a positive effect of a mentor as a regulatory agent and as a regulatory mechanism.

Five studies described the use of *learning plans*, in which students could record their goals, learning strategies and evidence of goal achievement.^34^, ^35^, ^37^, ^38^, ^40^ The use of learning plans helped the students as a framework and focus for learning and by increasing students' awareness of the learning process.^34^, ^35^ Furthermore, students were able to set a wide range of student‐determined learning objectives. Although this intervention was aimed at the complete SRL cycle, effects were only found in setting a wide range of learning objectives and in increased focus for learning (i.e. regulatory agent and mechanism).

The last group of interventions described the use of supportive tools such as an online environment^27^ and clinical encounter cards.^39^ The latter represent a tool for use in medical student feedback. Students can report their encounters in a structured way. The study did not clearly describe how these tools supported students' SRL and which effects were seen in the students.

## Discussion

In this systematic review, we aimed to obtain an overview of knowledge on SRL in medical students and residents in the clinical environment, and how this is influenced by the individual, the context and social interactions. Furthermore, we studied what interventions have been used to support the SRL in medical students and residents.

We found that a theoretical framework of SRL is often lacking, and when a theoretical framework was used (e.g. Zimmerman,^8^ Pintrich^32^ and Knowles^30^), this framework stemmed from research in a classroom setting. Considering the known influence of context on SRL, these models cannot be applied easily to the complex clinical context.

The studies of Berkhout et al*.,* 23 Woods et al.^24^ and Sagasser et al.^3^ described various patterns, approaches or activities that individuals use to self‐regulate their learning in the clinical environment. These different strategies suggest that there are not only individual differences between students' and residents' SRL strategies, but that the strategies they use also differ in different contexts. The patterns of Berkhout et al.^23^ and the approaches of Woods et al.^24^ largely match. In both studies, these patterns or approaches depend upon the degree to which students are able to adjust their behaviour to the clinical context and to what extent they show proactive behaviour in this. In addition, in the study performed by Sagasser et al*.,* 3 family practice residents picked different SRL strategies (short‐loop or long‐loop self‐regulation) depending on the complexity of the case. This suggests that the interaction between person and context has bearing on the strategies learners choose to apply in the clinical context.

There are many factors that, depending on the circumstances, may have either positive or negative influences on SRL. This supports earlier findings^2^, ^9^, ^42^ that learning in the clinical context is a complex process, in which there is an interplay of factors affecting the SRL process. Some of these factors are unique to the clinical environment (time pressure and patient‐related factors).^2^ A focus on stimulating positive influential factors and limiting negative factors might allow us to support learners' individual SRL needs and thereby improve SRL during clerkships and residency.

Self‐regulated learning is shaped by the interaction between the individual and the context. This review revealed individual differences between students' SRL strategies, implying that an individualised approach to supporting students in the clinical environment is required. The support of a mentor or coach and the use of learning plans and goal setting were found to be interventions that have a positive outcome on medical students' and residents' SRL processes. This might be caused by the fact that these interventions allow for adaptation to person and context. Moreover, the support of a coach or mentor seems to be a prerequisite for the SRL process to develop to its full potential.^2^, ^3^, ^26^, ^29^ All of the interventions revealed a positive outcome on the first two phases of SRL, namely goal setting and regulatory mechanisms. None of the studies focused on regulatory appraisals. This is the last phase of the SRL process, in which learners assess their progress towards goals. Regulatory appraisal is crucial to completing the learning experience and serves as a starting point for new learning endeavours. Therefore, more research is needed on the activities and interventions related to this last phase of SRL.

### Implications for practice

From our research, we conclude that SRL in the clinical context is not used to its full potential yet. In particular, assessing progress deserves more attention. Therefore, we recommend two parallel approaches: (i), introduce interventions to improve individuals' goal setting and reflection skills and improve their SRL confidence, and (ii), create a learning environment that gives students the opportunity to actually use their skills. This means that students should get, for example, more time for each patient, more patient encounters, support from their supervisor and guidance by a mentor.

### Strengths and limitations

One of the strengths of this systematic literature review is that we aimed to have a clear overview of the key strategies, the influential factors and effective interventions that stimulate the use of SRL in the clinical context. In doing so, we found gaps in the research, especially concerning the regulatory appraisal of the SRL process. This suggests that SRL is not used to its full potential yet.

This study has some limitations. First, we excluded articles in languages other than English or Dutch. That means that we were not able to include, for instance, cultural influences on SRL in our study. Second, only 18 studies were included in this review, many of which were observational. Third, we did not find, as we had expected, other relevant SRL interventions, such as portfolios or longitudinal integrated clerkships. A possible explanation may be that those interventions are often described within a different theoretical framework that was not included in our review. Future research on SRL should take these limitations into account.

### Implications for future research

To be able to apply the theoretical models reported in this review, we suggest further research to unravel the sub‐processes of SRL that are relevant to the clinical context in order to contribute to more elaborate SRL frameworks for this specific context. In addition, because of the mixed effects of some of the personal and contextual factors on SRL, we suggest more research on the interplay of these factors to unravel how SRL in the clinical environment can be strengthened.

## Conclusions

This study revealed that SRL in the clinical environment is a complex process and that there are individual differences in students' SRL. These are influenced by multiple factors at the individual, social and contextual levels. We were able to identify a knowledge gap in how learners assess their progress towards goals. These findings suggest that educators should adopt a dual approach when implementing SRL in the clinical context and should focus on both improving students' individual capacities to regulate their learning and creating a good SRL climate in the workplace, possibly supported by a mentor or coach.

## Contributors

MAvH‐S and ADD conceived the study idea. MAvH‐S, ADD, JJB and NvD contributed to the analysis. MDE, ADD, ADCJ and JJB contributed to the interpretation of the data. MAvH‐S drafted the work and all other authors helped revise it critically. All authors gave final approval of the submitted paper.

## Funding

none.

## Conflicts of interest

none.

## Ethical approval

not required.

## Supporting information


**Table S1.** Detailed description of the included studies.

**Table S2.** COREQ quality assessment (qualitative studies).

**Table S3.** MERSQI quality assessment (quantitative studies).

## References

[1] Sandars J . The use of reflection in medical education: AMEE guide no. 44. Med Teach 2009;31 (8):685–95. 19811204 10.1080/01421590903050374

[2] Berkhout JJ , Helmich E , Teunissen PW , van den Berg JW , van der Vleuten CP , Jaarsma AD . Exploring the factors influencing clinical students' self‐regulated learning. Med Educ 2015;49 (6):589–600. 25989407 10.1111/medu.12671

[3] Sagasser MH , Kramer AW , van der Vleuten CP . How do postgraduate GP trainees regulate their learning and what helps and hinders them? A qualitative study BMC Med Educ 2012;12:67. 22866981 10.1186/1472-6920-12-67 PMC3479408

[4] Boekaerts M . Self‐regulated learning: a new concept embraced by researchers, policy makers, educators, teachers, and students. Learn Instr 1997;7 (2):161–86.

[5] Zimmerman BJ . Attaining self‐regulation: a social cognitive perspective In: Boekaerts M, Pintrich P, Zeidner M, eds. Handbook of Self‐Regulation. New York, NY: Academic Press 2000;13–39.

[6] Pintrich PR . The role of motivation in promoting and sustaining self‐regulated learning. Int J Educ Res 1999;31 (6):459–70.

[7] Winne PH . Students' calibration of knowledge and learning processes: implications for designing powerful software learning environments. Int J Educ Res 2004;41 (6):466–88.

[8] Zimmerman BJ . Investigating self‐regulation and motivation: historical background, methodological developments, and future prospects. Am Educ Res J 2008;45 (1):166–83.

[9] Sitzmann T , Ely K . A meta‐analysis of self‐regulated learning in work‐related training and educational attainment: what we know and where we need to go. Psychol Bull 2011;137 (3):421–42. 21401218 10.1037/a0022777

[10] Bjork RA , Dunlosky J , Kornell N . Self‐regulated learning: beliefs, techniques, and illusions. Annu Rev Psychol 2013;64:417–44. 23020639 10.1146/annurev-psych-113011-143823

[11] Sandars J , Cleary TJ . Self‐regulation theory: applications to medical education: AMEE guide no. 58. Med Teach 2011;33 (11):875–86. 22022899 10.3109/0142159X.2011.595434

[12] Teunissen PW , Scheele F , Scherpbier AJ , van der Vleuten CP , Boor K , van Luijk SJ , van Diemen‐Steenvoorde JA . How residents learn: qualitative evidence for the pivotal role of clinical activities. Med Educ 2007;41 (8):763–70. 17661884 10.1111/j.1365-2923.2007.02778.x

[13] Swanwick T . Informal learning in postgraduate medical education: from cognitivism to ‘culturism'. Med Educ 2005;39 (8):859–65. 16048629 10.1111/j.1365-2929.2005.02224.x

[14] Meijerink AM , Boendermaker PM , Bolhuis S , Pols J . Een explorerend kwalitatief onderzoek naar het leren consultvoeren door huisartsen in opleiding. Tijdschr Med Onderwijs 2008;27 (1):5–13.

[15] Dornan T, Mann K, Scherpbier A, Spencer J, eds. Medical Education: Theory and Practice. London: Churchill‐Livingstone 2011.

[16] Berkhout JJ , Helmich E , Teunissen PW , van der Vleuten CP , Jaarsma AD . How clinical medical students perceive others to influence their self‐regulated learning. Med Educ 2017;51 (3):269–79. 27882583 10.1111/medu.13131 PMC5324607

[17] Driessen E , van Tartwijk J , Dornan T . The self critical doctor: helping students become more reflective. BMJ 2008;336 (7648):827–30. 18403547 10.1136/bmj.39503.608032.AD PMC2292362

[18] Tochel C , Haig A , Hesketh A , Cadzow A , Beggs K , Colthart I , Peacock H . The effectiveness of portfolios for post‐graduate assessment and education: BEME guide no. 12. Med Teach 2009;31 (4):299–318. 19404890 10.1080/01421590902883056

[19] Sharma R , Gordon M , Dharamsi S , Gibbs T . Systematic reviews in medical education: a practical approach: AMEE guide no. 94. Med Teach 2015;37 (2):108–24. 25314376 10.3109/0142159X.2014.970996

[20] Saks K , Leijen Ä . Distinguishing self‐directed and self‐regulated learning and measuring them in the E‐learning context. Procedia Soc Behav Sci 2014;112:190–8.

[21] Reed DA , Cook DA , Beckman TJ , Levine RB , Kern DE , Wright SM . Association between funding and quality of published medical education research. JAMA 2007;298 (9):1002–9. 17785645 10.1001/jama.298.9.1002

[22] Tong A , Sainsbury P , Craig J . Consolidated criteria for reporting qualitative research (COREQ): a 32‐item checklist for interviews and focus groups. Int J Qual Health Care 2007;19 (6):349–57. 17872937 10.1093/intqhc/mzm042

[23] Berkhout JJ , Teunissen PW , Helmich E , van Exel J , van der Vleuten CP , Jaarsma DA . Patterns in clinical students' self‐regulated learning behavior: a Q‐methodology study. Adv Health Sci Educ Theory Pract 2017;22 (1):105–21. 27235123 10.1007/s10459-016-9687-4 PMC5306423

[24] Woods NN , Mylopoulos M , Brydges R . Informal self‐regulated learning on a surgical rotation: uncovering student experiences in context. Adv Health Sci Educ Theory Pract 2011;16 (5):643–53. 21373808 10.1007/s10459-011-9285-4

[25] Turan S , Konan A . Self‐regulated learning strategies used in surgical clerkship and the relationship with clinical achievement. J Surg Educ 2012;69 (2):218–25. 22365869 10.1016/j.jsurg.2011.09.003

[26] Lockspeiser TM , Li ST , Burke AE et al. In pursuit of meaningful use of learning goals in residency: a qualitative study of pediatric residents. Acad Med 2016;91 (6):839–46. 26630605 10.1097/ACM.0000000000001015

[27] Alegria DA , Boscardin C , Poncelet A , Mayfield C , Wamsley M . Using tablets to support self‐regulated learning in a longitudinal integrated clerkship. Med Educ Online 2014;19:23638. 24646438 10.3402/meo.v19.23638 PMC3955768

[28] Aho JM , Ruparel RK , Graham E , Zendejas‐Mummert B , Heller SF , Farley DR , Bingener J . Mentor‐guided self‐directed learning affects resident practice. J Surg Educ 2015;72 (4):674–9. 25817011 10.1016/j.jsurg.2015.01.008 PMC4469518

[29] Nothnagle M , Anandarajah G , Goldman RE , Reis S . Struggling to be self‐directed: residents' paradoxical beliefs about learning. Acad Med 2011;86 (12):1539–44. 22030764 10.1097/ACM.0b013e3182359476

[30] Knowles M . Self‐Directed Learning: A Guide for Learners and Teachers. Englewood Cliffs, NJ: Cambridge Adult Education 1975.

[31] Artino AR Jr , Dong T , DeZee KJ , Gilliland WR , Waechter DM , Cruess D , Durning SJ . Achievement goal structures and self‐regulated learning: relationships and changes in medical school. Acad Med 2012;87 (10):1375–81. 22914521 10.1097/ACM.0b013e3182676b55

[32] Pintrich P . The role of goal orientation in self‐regulated learning In: Boekaerts M, Zeidner M, Pintrich PR, eds. Handbook of Self‐Regulation. San Diego, CA: Academic Press 2000;451–502.

[33] Loyens S , Magda J , Rikers R . Self‐directed learning in problem‐based learning and its relationships with self‐regulated learning. Educ Psychol Rev 2008;20:411–27.

[34] Stuart E , Sectish TC , Huffman LC . Are residents ready for self‐directed learning? A pilot program of individualized learning plans in continuity clinic. Ambul Pediatr 2005;5 (5):298–301. 16167854 10.1367/A04-091R.1

[35] Smith SJ , Kakarala RR , Talluri SK , Sud P , Parboosingh J . Internal medicine residents' acceptance of self‐directed learning plans at the point of care. J Grad Med Educ 2011;3 (3):425–8. 22942979 10.4300/JGME-03-03-30 PMC3179229

[36] George P , Reis S , Dobson M , Nothnagle M . Using a learning coach to develop family medicine residents' goal‐setting and reflection skills. J Grad Med Educ 2013;5 (2):289–93. 24404275 10.4300/JGME-D-12-00276.1 PMC3693696

[37] Li ST , Favreau MA , West DC . Pediatric resident and faculty attitudes toward self‐assessment and self‐directed learning: a cross‐sectional study. BMC Med Educ 2009;9:16. 19364401 10.1186/1472-6920-9-16 PMC2673219

[38] Smith P , Morrison J . Clinical clerkships: students can structure their own learning. Med Educ 2006;40 (9):884–92. 16925639 10.1111/j.1365-2929.2006.02546.x

[39] Tolsgaard MG , Arendrup H , Pedersen P , Ringsted C . Feasibility of self‐directed learning in clerkships. Med Teach 2013;35 (8):e1409–15. 23444885 10.3109/0142159X.2013.770135

[40] Li ST , Tancredi DJ , Co JP , West DC . Factors associated with successful self‐directed learning using individualized learning plans during pediatric residency. Acad Pediatr 2010;10 (2):124–30. 20206911 10.1016/j.acap.2009.12.007

[41] Li ST , Paterniti DA , Co JP , West DC . Successful self‐directed lifelong learning in medicine: a conceptual model derived from qualitative analysis of a national survey of pediatric residents. Acad Med 2010;85 (7):1229–36. 20592521 10.1097/ACM.0b013e3181e1931c

[42] Brydges R , Butler D . A reflective analysis of medical education research on self‐regulation in learning and practice. Med Educ 2012;46 (1):71–9. 22150198 10.1111/j.1365-2923.2011.04100.x

