# Supplementary material for: Self‐regulated learning in the clinical context: a systematic review
Source: Med Educ. 2018 Jun 25;52(10):1008–15. doi: 10.1111/medu.13615 (PMC6175376; doi:10.1111/medu.13615)
Supplement: Supplementary file 1 — Table S1. Detailed description of the included studies. [file MEDU-52-1008-s001.docx]

| *Table S1. Detailed description of the included studies* | | | | | | | |
| --- | --- | --- | --- | --- | --- | --- | --- |
| Source | **Title** | **Journal** | **Study design & data collection** | **Study population** | **Study location** | **Sample size** | **Brief abstract** |
| Aho et al. (2015)^28^ | Mentor-Guided Self-Directed Learning Affects Resident Practice | Journal of Surgical Education | Cross-sectional study  Intervention: mentor-guided 6 week minimally invasive surgery (MIS) rotation  Evaluation: three meetings with staff surgeon, post-practice survey | Postgraduate year 2 general surgery residents | United States | 12 general surgery residents | This study identified that mentor-guided SDL stimulated surgical residents to practice laparoscopic skills with greater frequency in the simulation laboratory and this subsequently led to improved MIS skills without significantly increasing the need for faculty-led instruction. |
| Alegría et al. (2014)^27^ | Using tablets to support self-regulated learning in a longitudinal integrated clerkship | Medical Education Online | Qualitative study  Intervention: use of tablet computers  Evaluation: focus groups | Longitudinal integrated clerkship (LIC) students | United States | 15 LIC students | This study described the use of tablets as a learning tool to support students’ self-regulated learning. |
| Artino et al. (2012)^31^ | Achievement Goal Structures and Self-Regulated Learning: Relationships and Changes in Medical School | Academic Medicine | Cohort study | Students at different phases of medical school training | United States (Bethesda) | 304 medical students  (87 first-year; 88 second year; 64 third-year; 65 fourth-year students) | This study explored how medical students’ perceptions of the learning environment related to their self-regulated learning behaviours and how students’ perceptions and behaviours correlated with performance and change across medical school. |
| Berkhout et al. (2015)^2^ | Exploring the factors influencing clinical students’ self-regulated learning | Medical Education | Qualitative study (constructivist grounded theory)  Semi-structured interviews | Medical students enrolled in clerkships | Netherlands | 17 medical students | This study described the factors influencing the process of self-regulated learning of medical students in the clinical environment. They found that the factors influencing this process are affected by personal, social and contextual attributes. |

| Source | Title | Journal | Study design & data collection | Study population | Study location | Sample size | Brief abstract |
| --- | --- | --- | --- | --- | --- | --- | --- |
| Berkhout et al. (2016)^23^ | Patterns in clinical students’ self-regulated learning behavior: A Q-methodology study | Advances in Health Sciences Education | Q-methodology study (qualitative and quantitative methods)  Evaluation of statements on SRL behaviours | Undergraduate medical students in the clinical phase | Netherlands | 74 medical students | This study identified five patterns in students’ SRL behaviour in the clinical environment (Engaged, Critically opportunistic, Uncertain, Restrained and Effortful). |
| George et al. (2013)^36^ | Using a Learning Coach to Develop Family Medicine Residents’ Goal Setting and Reflection Skills | Journal of Graduate Medical Education | Mixed method study  Intervention: monthly meetings with learning coach and the use of an electronic portfolio  Evaluation: learning coach notes/ observations, portfolio entries, semi-structured interviews. | Second year family practice residents | Rhode Island (United States) | 25 residents | This study described the effect of monthly meetings with a learning coach and the use of an electronic portfolio for promoting the development of SDL skills. The outcome of the intervention was that it helped residents to identify learning needs, set learning goals, and reflect on their learning. |
| Li et al. (2009)^37^ | Pediatric resident and faculty attitudes toward self-assessment and self-directed learning: a cross-sectional study | BMC Medical Education | Cross-sectional study  Paper-based (for residents) and web-based survey (for faculty members).  Respondents needed to rate their attitudes, knowledge, SDL and individualized learning plans (ILPs) on a 6-point Likert scale. The free text responses were qualitatively analysed | Paediatric residents and faculty members at a single institution | United States | 36 residents (12 first-year and 24 senior residents)  43 faculty members | The aim of this study was to compare faculty and resident attitudes, knowledge and skills about self-assessment, SDL and ILPs. They also compared first-year and senior residents’ opinions. |

| Source | Title | Journal | Study design & data collection | Study population | Study location | Sample size | Brief abstract |
| --- | --- | --- | --- | --- | --- | --- | --- |
| Li et al. (2010)^41^ | Successful Self-Directed Lifelong Learning in Medicine: A Conceptual Model Derived from Qualitative Analysis of a National Survey of Pediatric Residents | Academic Medicine | Cross-sectional study  Survey with questions about residents’ experiences with individualized learning plans (ILPs) (barriers and strategies for achieving goals) | Paediatric and medicine/ paediatric residents from all years | United States | 992 residents | This study proposes, on the basis of the data, a conceptual model for self-directed lifelong learning. In this model, they involved the creation of learning goals and plan development based on individual reflection and self-assessment, and continual revision of goals and/or plans based on degree of goal attainment. |
| Li et al. (2010)^40^ | Factors Associated with Successful Self-Directed  Learning Using Individualized Learning Plans  During Pediatric Residency | Academic Pediatrics | Cross-sectional study  Web-based survey; outcomes were measured on a 5-point Likert scale | paediatric and medicine/  paediatric residents from all years and program directors | United States | 46 program directors  992 residents | This study described the analyses of a web-based survey. The primary outcome was resident self-report of average progress toward achieving learning goals for their most recent ILP. The secondary outcome was resident self-report of progress toward their most important learning goal in that ILP. This study concluded that the most important factors associated with effective self-directed learning were resident characteristics. |
| Lockspeiser et al. (2016)^26^ | In Pursuit of Meaningful Use of Learning Goals in Residency: A qualitative study of paediatric residents | Academic Medicine | Qualitative study  Brief interviews and focus groups | Program directors of paediatric residency and third-year (senior) paediatric residents | United States | 12 program directors  95 senior residents | This study identified five aspects of the learning environment that influence the meaningful use of learning goals in residency. The themes that emerged demonstrated all three phases of SRL. |
| Nothnagle et al. (2011)^29^ | Struggling to Be Self-Directed: Residents’ Paradoxical Beliefs About Learning | Academic Medicine | Qualitative study  In-depth, semi-structured interviews | Final-year family medicine residents | Rhode Island (United States) | 13 residents | The aim of this study was to examine the prevailing culture of learning in residency, residents’ approaches to learning, and their views on SDL. The study concluded that graduating residents lacked confidence in their SDL skills and their ability to manage their learning, especially in clinical settings. |
| Source | **Title** | **Journal** | **Study design & data collection** | **Study population** | **Study location** | **Sample size** | **Brief abstract** |
| Sagasser et al. (2012)^3^ | How do postgraduate GP trainees regulate their learning and what helps and hinders them? A qualitative study | BMC Medical Education | Qualitative (phenomenological approach)  Semi-structured interviews | First- and third-year general practice trainees | Netherlands | 10 first-year residents  11 third-year residents | The aim of this study was to explore how GP trainees regulate their learning in the workplace, how external regulation promotes SRL and which elements facilitate or impede self-regulation and learning. They found two main themes: 1) self-regulation loops and 2) elements influencing self-regulation. |
| Smith et al. (2006)^38^ | Clinical clerkships: students can structure their own learning | Medical Education | Cross-sectional study | Undergraduate medical students who were allocated a 5-week clerkship in general practice | United Kingdom (Glasgow) | 143 medical students | The aim of this study was to assess whether student-determined learning objectives may help students to structure their learning. They found that this method allowed students to address gaps in their knowledge in a clerkship where teaching is largely based on opportunistic contact. |
| Smith et al. (2011)^35^ | Internal Medicine Residents’ Acceptance of Self-Directed Learning Plans at the Point of Care | Journal of Graduate Medical Education | Cross-sectional study  Intervention: formulating self-directed learning plans (SDLPs)  Evaluation: questionnaire | Internal medicine residents | Michigan (United States) | 26 residents | This study described residents’ perceptions and attitudes regarding the use of SDLPs. |
| Stuart et al. (2005)^34^ | Are Residents Ready for Self-Directed learning? A Pilot Program of Individualized Learning Plans in Continuity Clinic | Ambulatory Pediatrics | Cohort study  Intervention: implementation of a pilot program using ILPs.  Evaluation: learning plan program questionnaire | Faculty members with their continuity residents (paediatric) | United States | 13 faculty members  42 paediatric residents | The aim of this study was to explore residents’ and faculty members’ reactions to using ILPs when ILPs were offered as an optional educational tool. The study described the perceived benefits and barriers to using ILPs. |

| Source | Title | Journal | Study design & data collection | Study population | Study location | Sample size | Brief abstract |
| --- | --- | --- | --- | --- | --- | --- | --- |
| Tolsgaard et al. (2013)^39^ | Feasibility of self-directed learning in clerkships | Medical Teacher | Mixed method study  Pilot testing and focus groups for the evaluation of the usefulness of clinical encounter-cards (CEC) to learning in clerkships  Intervention: implementation of CEC in clerkship  Evaluation: online survey using a nine-point Likert-scale | Year-four and year-five medical students | Denmark | Pilot testing/ focus group:  8 year-four and 11 year-five medical students  Cohort study: 99 year-four and 113 year-five students | This study described if the use of clinical encounter-cards useful is to learning in clerkship. They first started with two pilot groups to explore the feasibility and usefulness of the CECs. They subsequently introduced the CECs in two cohorts of students in clinical clerkships. They found that the self-directed CECs can have a positive effect on participation and clinical reasoning when used by motivated students and supported by medical educators. However, participatory practice was limited in clinical clerkships. |
| Turan et al. (2012)^25^ | Self-Regulated Learning Strategies Used in Surgical Clerkship and the Relationship with Clinical Achievement | Journal of Surgical Education | Cross-sectional study  Evaluation: motivated strategies for learning questionnaire (MSLQ) mid-clerkship, and case-based examination, objective structured clinical examination (OSCE), and tutor evaluations at the end of clerkship | Fourth year medical students who continued to a general surgery clerkship | Turkey | 273 medical students | This study described the self-regulated learning strategies used by medical students in surgical clerkship and their relation with clinical achievement. The findings showed that students use self-regulated learning skills at medium levels during their surgery clerkship. Furthermore, they observed that a greater self-efficacy for learning resulted in higher OSCE scores. No significant relationship was defined between self-regulated learning skills and case-based examination scores. |

| Source | Title | Journal | Study design & data collection | Study population | Study location | Sample size | Brief abstract |
| --- | --- | --- | --- | --- | --- | --- | --- |
| Woods et al (2011)^24^ | Informal self-regulated learning on surgical rotation: uncovering student experiences in context | Advances in Health Sciences Education | Qualitative study  Focus groups | Third and fourth year medical students | Toronto, Canada | 313 medical students | The aim of this study was to examine undergraduate medical students’ day-to-day learning strategies in order to better understand the process of informal SRL in practice. Participants described four key learning activities and thematic analyses of the focus groups revealed three distinct learning approaches: acquiescing to a perceived lack of learning opportunities, choosing from available learning opportunities, and creating new learning opportunities. |
