# Supplementary material for: Self‐regulated learning in the clinical context: a systematic review
Source: Med Educ. 2018 Jun 25;52(10):1008–15. doi: 10.1111/medu.13615 (PMC6175376; doi:10.1111/medu.13615)
Supplement: Supplementary file 2 — Table S2. COREQ quality assessment (qualitative studies). [file MEDU-52-1008-s002.docx]

|  | *Table S2. COREQ quality assessment (qualitative studies)* | | | | | | | |
| --- | --- | --- | --- | --- | --- | --- | --- | --- |
|  | *Domain 1: research team and reflexivity* | | *Domain 2: study design* | | | | *Domain 3: analysis and findings* | |
| **Study** | **Personal characteristics** | **Relationship with participants** | **Theoretical framework** | **Participant selection** | **Setting** | **Data collection** | **Data analysis** | **Reporting** |
| Alegría et al. (2014)^27^ | - | - | + | + | - | + | + | + |
| Berkhout et al. (2015)^2^ | + | +/- | + | + | +/- | + | + | + |
| Lockspeiser et al. (2016)^26^ | + | - | + | + | - | + | + | + |
| Nothnagle et al. (2011)^29^ | + | + | - | + | +/- | + | + | + |
| Sagasser et al. (2012)^3^ | + | +/- | + | +/- | +/- | + | + | + |
| Woods et al. (2011)^24^ | - | + | + | + | + | +/- | + | + |
| **Mixed methods studies** | | | | | | | | |
| George et al. (2013)^36^ | + | + | + | +/- | - | - | +/- | +/- |
| Tolsgaard et al. (2013)^39^ | +/- | - | + | + | - | - | + | + |
