# Supplementary material for: Self‐regulated learning in the clinical context: a systematic review
Source: Med Educ. 2018 Jun 25;52(10):1008–15. doi: 10.1111/medu.13615 (PMC6175376; doi:10.1111/medu.13615)
Supplement: Supplementary file 3 — Table S3. MERSQI quality assessment (quantitative studies). [file MEDU-52-1008-s003.docx]

|  | *Table S3. MERSQI – POINTS PER ITEM* | | | | | | | |  |
| --- | --- | --- | --- | --- | --- | --- | --- | --- | --- |
|  | *Domain 1* | *Domain 2* | | *Domain 3* | *Domain 4* | *Domain 5* | | *Domain 6* |  |
| **Study** | **Study design (max 3)** | **Sampling: institutions**  **(max 3)** | **Sampling: response rate**  **(max 3)** | **Type of data**  **(max 3)** | **Validity evidence for evaluation instrument scores (max 3)** | **Data analysis: sophistication**  **(max 3)** | **Data analysis: appropriate**  **(max 3)** | **Outcome**  **(max 3)** | **Total score**  **(max 18)** |
| Aho et al. (2015)^28^ | 1 | 0.5 | 1.5 | 1 | 1 | 2 | 1 | 1.5 | **9,5** |
| Artino et al. (2012)^31^ | 2 | 0.5 | 0.5 | 1 | 3 | 2 | 1 | 1 | **11** |
| Berkhout et al. (2016)^23^ | 1 | 0.5 | 0.5 | 1 | 1 | 2 | 1 | 1 | **8,0** |
| Li et al. (2009)^37^ | 1 | 0.5 | 1.5 | 1 | 1 | 2 | 1 | 1 | **9** |
| Li et al. (2010)^41^ | 1 | 1.5 | 1 | 1 | 1 | 2 | 1 | 1 | **9,5** |
| Li et al. (2010)^40^ | 1 | 1.5 | 1 | 1 | Not applicable | 1 | 1 | 1 | **7,5** |
| Smith et al. (2006)^38^ | 1 | 0.5 | 1.5 | 1 | 1 | 1 | 1 | 1 | **8,0** |
| Smith et al. (2011)^35^ | 1 | 0.5 | 1 | 1 | 1 | 1 | 1 | 1 | **7,5** |
| Stuart et al. (2005)^34^ | 2 | 0.5 | 1 | 1 | 1 | 1 | 1 | 1 | **8,5** |
| Turan et al. (2012)^25^ | 1 | 0.5 | 1.5 | 1 | 3 | 2 | 1 | 1 | **11** |
| **Mixed-methods studies** | | | | | | | | | |
| George et al. (2013)^36^ | 1.5 | 0.5 | 1 | 3 | 2 | 2 | 1 | 1 | **12** |
| Tolsgaard et al. (2013)^39^ | 2 | 0.5 | 1 | 1 | 1 | 2 | 1 | 1 | **9,5** |
